# The Galaxy platform for accessible, reproducible and collaborative biomedical analyses: 2020 update

**DOI:** 10.1093/nar/gkaa434

**Published:** 2020-06-01

**Authors:** Vahid Jalili, Enis Afgan, Qiang Gu, Dave Clements, Daniel Blankenberg, Jeremy Goecks, James Taylor, Anton Nekrutenko

**Affiliations:** Department of Biomedical Engineering, Oregon Health & Science University, Portland, OR, USA; Department of Biology, Johns Hopkins University, Baltimore, MD, USA; Department of Biomedical Engineering, Oregon Health & Science University, Portland, OR, USA; Department of Biology, Johns Hopkins University, Baltimore, MD, USA; Genomic Medicine Institute, Lerner Research Institute, Cleveland Clinic, Cleveland, OH, USA; Department of Biomedical Engineering, Oregon Health & Science University, Portland, OR, USA; Department of Biology, Johns Hopkins University, Baltimore, MD, USA; Department of Biochemistry and Molecular Biology, Penn State University, University Park, PA, USA

## Abstract

Galaxy (https://galaxyproject.org) is a web-based computational workbench used by tens of thousands of scientists across the world to analyze large biomedical datasets. Since 2005, the Galaxy project has fostered a global community focused on achieving accessible, reproducible, and collaborative research. Together, this community develops the Galaxy software framework, integrates analysis tools and visualizations into the framework, runs public servers that make Galaxy available via a web browser, performs and publishes analyses using Galaxy, leads bioinformatics workshops that introduce and use Galaxy, and develops interactive training materials for Galaxy. Over the last two years, all aspects of the Galaxy project have grown: code contributions, tools integrated, users, and training materials. Key advances in Galaxy's user interface include enhancements for analyzing large dataset collections as well as interactive tools for exploratory data analysis. Extensions to Galaxy's framework include support for federated identity and access management and increased ability to distribute analysis jobs to remote resources. New community resources include large public servers in Europe and Australia, an increasing number of regional and local Galaxy communities, and substantial growth in the Galaxy Training Network.

## INTRODUCTION

Biomedical studies have become data-intensive, with ever evolving technological and computational demands. Increasing reliance on complex computational methods prevents many biomedical researchers, from accessing and making effective use of these datasets and methods. This also presents significant barriers to reproducibility, dissemination and generalized reuse. Since 2005, the Galaxy project (https://galaxyproject.org) has provided free and open solutions to address these considerable barriers in biomedical research. Galaxy is an open source, community-driven, and web-based platform for accessible, reproducible, and transparent computational research and training. Galaxy supports accessibility by enabling complex computational analysis to be performed from a web browser without requiring programming experience or training in high performance computing. Reproducibility is ensured, as Galaxy automatically captures execution information (e.g. tool name, version, inputs, outputs and parameters) so that a user doesn’t have to manually track provenance; hence, any user can repeat and understand a complete computational analysis, from tool parameters to the dependency tree. Galaxy users are able to share and publish their exact analysis histories, results, workflows and visualizations directly over the web, enabling transparency of computational research efforts and artifacts.

The Galaxy software ecosystem consists of multiple components: (a) an integrated repository of tools for a wide-range of biomedical studies including sequence and variant analysis, metagenomics, proteomics, and transcriptomics (1); (b) a web application that enables exploratory data analysis using the integrated tools via a web interface; (c) a multitude of specialized installations of the web application (e.g. https://usegalaxy.org for biomedical research, see https://galaxyproject.org/use/ for a complete list of the installations); (d) a training network that provides tutorials and organizes workshops on using Galaxy for different studies (https://galaxyproject.org/learn/) and (e) An inclusive and diverse community of developers, educators, and researchers encompassing a wide range of skill sets, scientific domains, and research practices that provide development and support (https://help.galaxyproject.org; the adopted *code of conduct* is available at https://github.com/galaxyproject/galaxy/blob/dev/CODE_OF_CONDUCT.md).

In the past year, the Galaxy project has seen major growth as a platform, a resource, and a community. The usegalaxy.* alliance operates large Galaxy deployments in the US (usegalaxy.org), Europe (usegalaxy.eu) and Australia (usegalaxy.org.au). The Galaxy framework has been widely deployed by others, with 125 other known public instances (https://galaxyproject.org/use). The developer community has thrived, with >7500 tools contributed to the Galaxy ToolShed as of January 2020.

Genomics research is continuously evolving and current challenges include the rapid growth in size and complexity of new datasets, the continuing expansion in the breadth of research areas capable of generating high throughput data, and the integration of genomics with other molecular and phenotypic data. In this article, we describe the latest advances in the Galaxy platform designed to address these challenges.

This manuscript describes work performed by a large group of people located around the world with complementary skills who are critical to the success of the Galaxy project. These individuals are listed in Table 1.

**Table 1.** Members of the four major regional Galaxy teams–Africa, Australia, Europe and North America

Region	Members	Affiliation
Africa	Christopher Barnett, Tharindu Senapathi	Chemistry Department and Scientific Computing Research Unit at the University of Cape Town
	Thoba Lose, Ziphozakhe Mashologu, Peter van Heusden	South African National Bioinformatics Institute, University of the Western Cape, South Africa
Australia	Catherine Bromhead, Simon Gladman, Nuwan Goonasekera, Christina Hall, Andrew Lonie	Melbourne Bioinformatics, University of Melbourne, Melbourne, Victoria, Australia
	Maria Doyle	Peter MacCallum Cancer Centre, Melbourne, Victoria, Australia
	Thom Cuddihy, Igor Makunin, Gareth Price, Nick Rhodes, Michael Thang	QFAB Bioinformatics, QCIF, Brisbane, Queensland, Australia
Europe	Loraine Brillet-Guéguen, Gildas Le Corguillé	ABiMS, Roscoff, France
	Christophe Antoniewski	ARTbio, CNRS and Sorbonne Université, Paris France
	Léa Bellenger	ARTbio, INSERM and Sorbonne Université, Paris, France
	Naïra Naouar	ARTbio, Sorbonne Université, Paris, France
	Nadia Goué	AuBi, Mesocentre, Clermont Auvergne University, France
	Saskia Hiltemann, Youri Hoogstrate, Bas Horsman, Rick Jansen, Yunlei Li, Andrew Stubbs, David van Zessen	Bioinformatics, Erasmus MC Cancer Institute, Rotterdam, Netherlands
	Frederik Coppens, Bert Droesbeke, Ignacio Eguinoa, Michiel Van Bel	Center for Plant Systems Biology, Vlaams Instituut voor Biotechnologie, Ghent, Belgium
	Jean-François Dufayard, Maryline Summo	CIRAD, Montpellier, France
	Anshu Bhardwaj	CSIR-Institute of Microbial Technology, France
	Tomas Klingström	Department of Animal Breeding and Genetics, Swedish University of Agricultural Sciences, Uppsala, Sweden
	Federico Zambelli	Department of Biosciences, University of Milan, Milano, Italy
	Rolf Backofen, Bérénice Batut, Simon Bray, Gianmauro Cuccuru, Anika Erxleben, Stephan Flemming, Björn Grüning, Alireza Khanteymoori, Anup Kumar, Jan Leendertse, Wolfgang Maier, Helena Rasche, Mehmet Tekman, Joachim Wolff, Oleg Zharkov	Department of Computer Science, Albert-Ludwigs-University Freiburg, Freiburg, Germany
	Anne Fouilloux	Department of Geosciences, University of Oslo, Norway
	Florence Combes, Yves Vandenbrouck	Department of Health, CEA, Grenoble, France
	Nicola Soranzo	Earlham Institute, Norwich Research Park, Norwich, UK
	Lucille Lopez-Delisle	EPFL SV ISREC UPDUB, 1015 Lausanne, Switzerland
	Pablo Moreno	European Bioinformatics Institute (EMBL-EBI)
	Hans-Rudolf Hotz	Friedrich Miescher Institute for Biomedical Research, Basel, Switzerland
	Sarah Maman	GenPhySE, Université de Toulouse, INRA, INPT, ENVT, Castanet Tolosan, France
	Matthias Bernt	Helmholtz Centre for Environmental Research, UFZ, Young Investigators Group Bioinformatics and Transcriptomics, Leipzig, Germany
	Anthony Bretaudeau	IGEPP, INRAE, Institut Agro, Univ Rennes, Rennes, France
	Timothy Dudgeon	Informatics Matters Ltd.
	Olivier Inizan, Valentin Loux	INRAE, Jouy-en-Josas, France
	Kenzo-Hugo Hillion, Valentin Marcon, Fabien Mareuil, Hervé Ménager, Rémi Planel	Institut Pasteur, Paris, France
	Marco Antonio Tangaro	Institute of Biomembranes, Bioenergetics and Molecular Biotechnologies, National Research Council, Bari, Italy
	Alexis Dereeper	Institute of Research for Development, Marseille, France
	Melanie Föll	Institute of Surgical Pathology, Medical Center, Albert-Ludwigs-University Freiburg, Freiburg, Germany
	Peter Cock	James Hutton Institute, UK
	Peter Selten	KWS SAAT SE & Co. KGaA
	Ruben Vorderman	Leiden University Medical Center, Netherlands
	Alan Amossé, Yvan Le Bras, Coline Royaux	Museum National d’Histoire Naturelle, Paris, France
	Franck Giacomoni	PFEM, INRAE, Saint Genès Champanelle, France
	Thanh Le-Viet, Andrew Page	Quadram Institute Bioscience, Norwich Research Park, Norwich, UK
	Thomas Lawson	School of Biosciences, University of Birmingham, UK
	Olivier Sallou	Univ Rennes, Inria, CNRS, IRISA, Rennes France
	Ralf Weber	University of Birmingham, UK
	Krzysztof Poterlowicz	University of Bradford, UK
	Ivan Kuzmin	University of Tartu, Estonia
North America	Dan Fornika	BC Centre for Disease Control, Canada
	Carrie Ganote	Bioinformatics Analyst at Indiana University, USA
	Dave Bouvier, Martin Čech, John Chilton, Nate Coraor, Assunta DeSanto, Jennifer Hillman-Jackson, Kaivan Kamali, Nick Keener, Delphine Lariviere, Anton Nekrutenko, Nick Stoler, Marius van den Beek	Department of Biochemistry and Molecular Biology, Penn State University, University Park, PA, USA
	Enis Afgan, Dannon Baker, Dave Clements, Sergey Golitsynskiy, Juleen Graham, Aysam Guerler, Mohammad Heydarian, Alexandru Mahmoud, Alex Ostrovsky, Nathan Roach, James Taylor, Jenn Vessio	Department of Biology, Johns Hopkins University, Baltimore, MD, USA
	Jeremy Goecks, Qiang Gu, Mason Houtz, Vahid Jalili, Luke Sargent	Department of Biomedical Engineering, School of Medicine, Oregon Health and Science University, Portland, OR, USA
	Michael Schatz	Dept. of Computer Science and Biology, Johns Hopkins University, Baltomore, MD, USA
	Daniel Blankenberg, Jayadev Joshi, Vijay Nagampalli	Genomic Medicine Institute, Lerner Research Institute, Cleveland Clinic, Cleveland, OH, USA
	Greg Von Kuster	Huck Institutes of the Life Sciences, Penn State University, University Park, PA, USA
	Robert Leach, Lance Parsons	Lewis-Sigler Institute of Integrative Genomics, Princeton University, USA
	Brad Langhorst	New England Biolabs, USA
	Philip Mabon, Aaron Petkau, Jeffrey Thiessen	Public Health Agency of Canada, Canada
	Arthur Eschenlauer, Tim Griffin, Pratik Jagtap, James Johnson, Praveen Kumar, Subina Mehta	University of Minnesota, Minnesota, Minneapolis, MN, USA

## NEW AND ENHANCED FEATURES

### Enhanced Galaxy user experience for increasingly complex analysis

Reduction in sequencing cost has led to increases in the complexity and size of DNA sequencing data. One of the major goals of Galaxy is to allow users to upload, organize, and manipulate complex experimental designs entirely through the Galaxy user interface (UI). To achieve this objective, we developed a number of new features described below.

#### Dataset collections for analysis of unlimited number of datasets through UI

**Figure 1.**
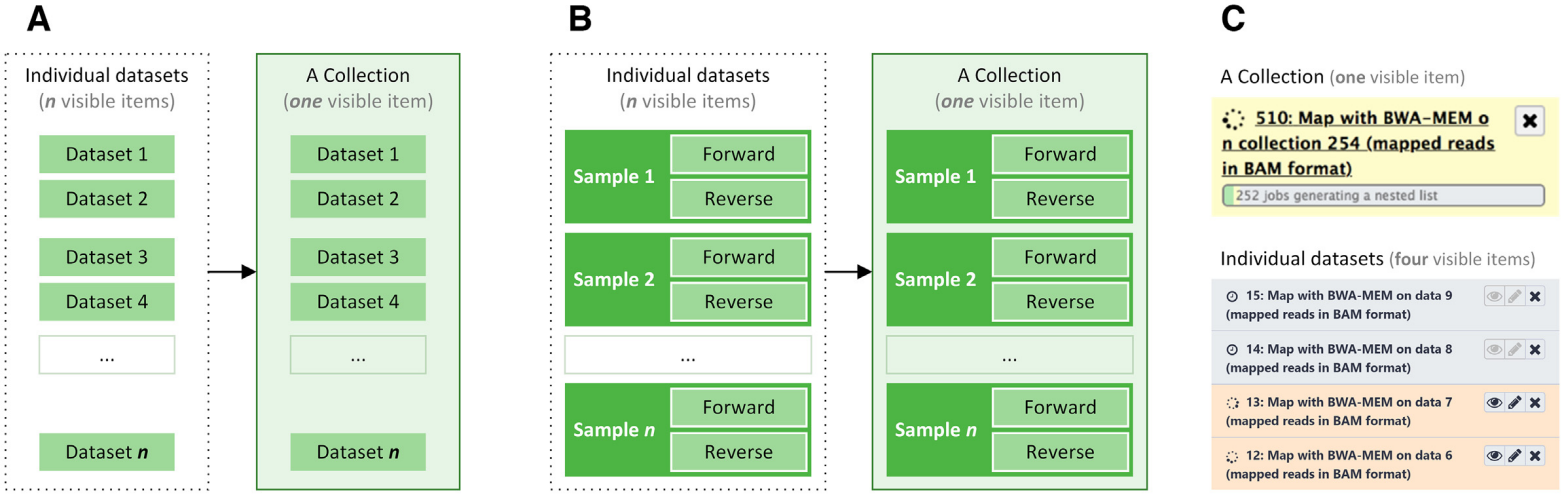
Examples of datasets collections. Panel **A** illustrates a simple list of n datasets that are encapsulated as a single collection item. Panel **B** illustrates *n* samples with nested relation (datasets of forward and reverse reads) represented as a collection item. Panel **C** is a screenshot of a collection and dataset items in Galaxy's history. Here, BWA-MEM (19) is generating 254 BAM datasts, which can be represented as a collection, or 254 individual datasets items (four shown here).

Modern experiments typically involve a large number of datasets organized as complex hierarchies. For example, consider the simple case of a resequencing experiment with 10 samples composed of 20 files, corresponding to 10 *forward* and 10 *reverse* paired-end read datasets. Representing these individual datasets as 20 interface elements without their intrinsic hierarchical relationship is counter-intuitive and impractical. Therefore, we have developed the concept of *dataset collections* for representing complex assemblies of datasets, which enable Galaxy users to encode semantic relationships. Figure 1 illustrates collections representing either a simple list of datasets (Figure 1 panel A) or a set of samples from a paired-end library (Figure 1, panel B; a list of pairs).

#### Name tags and group tags

**Figure 2.**
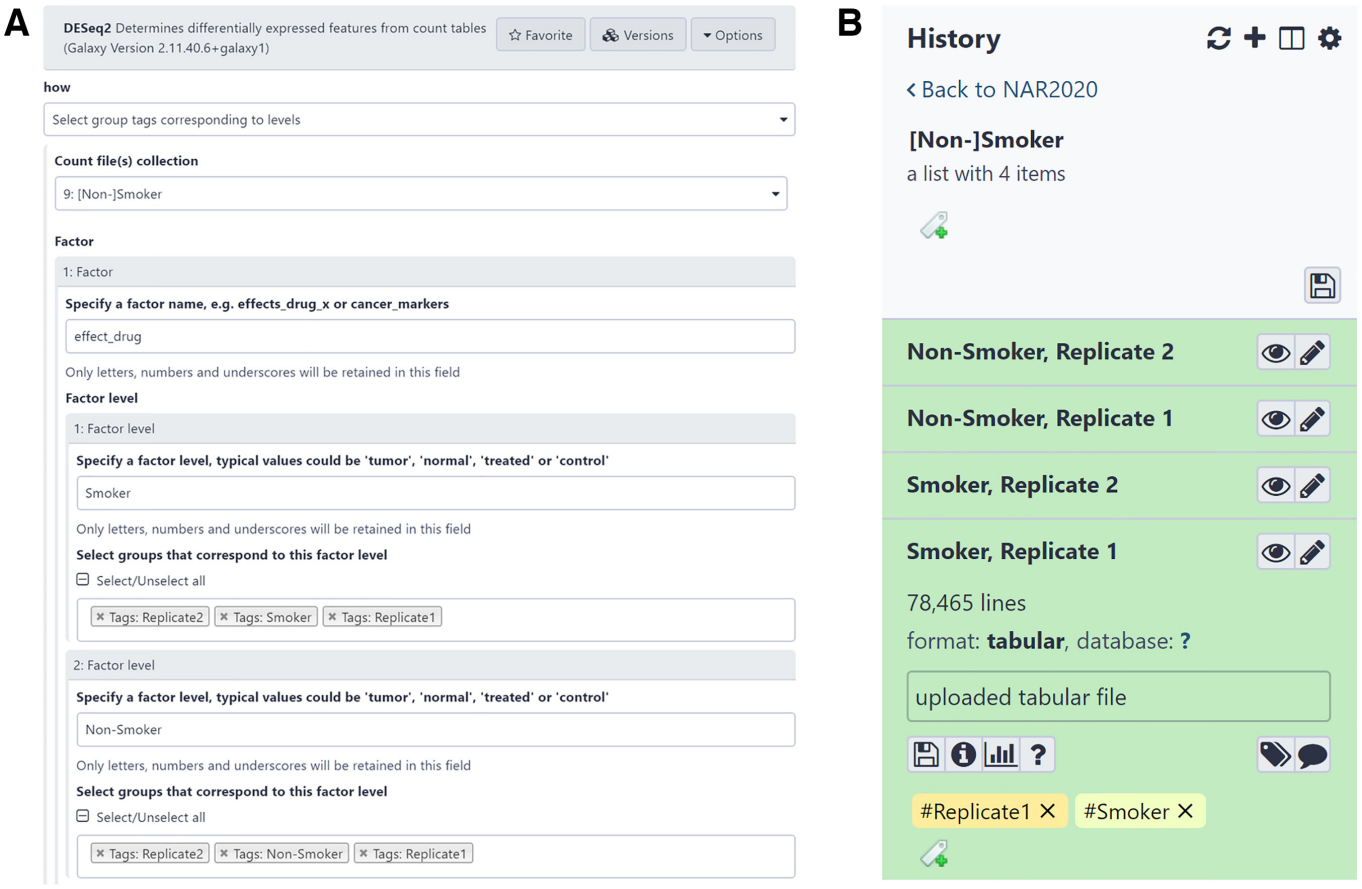
The figure illustrates a way for expressing hierarchical dataset relationships in Galaxy and use them in tools. Panel B shows four datasets representing two conditions (i.e. smoker and non-smoker) each with two replicates, organized under [Non-]Smoker collection using Galaxy's Collection Builder (the rules used for building this collection are given in Supplementary Material). These datasets are assigned with group tags (e.g. Smoker and Replicate 1 as shown in the figure), which can then be used to simplify their selection as inputs for tools. Panel A illustrates specifying these datasets as inputs for DESeq2 (2).

To allow users to easily follow steps within Galaxy analyses (*histories*) we have developed *name tags*, which allow users to see all analysis steps that use or derive from a given dataset, and power new features for multiple factor analysis of collections of datasets. G*roup tags* are a special class of tags with key-value pairs that can be attached to a collection during upload or using collection operation tools. These tags can describe multiple sets of variables for a collection. Once set, these tags can be used intelligently by tools that need to divide collections into multiple overlapping factors or sets of datasets. Figure 2 illustrates an example of using *group tags* to study datasets that differentiate gene expression between *smokers* and *non-smokers*. The Galaxy tool interface for DESeq2 (2), a popular tool for gene expression data analysis, is able to use *group tags* to allow comparisons between factors such as, in this case, *smokers* and *non-smokers*.

#### Data selector dialog

Previously, selecting datasets for analysis could be time-consuming in Galaxy, due to potentially having thousands of datasets available to a user, and which may be distributed across many individual analysis *histories* and shared *libraries*. Galaxy data *libraries* are repositories for shared datasets. To simplify dataset selection, a new dataset selector dialog has been implemented in tool forms, visualizations, and wherever datasets are used. It allows selection of datasets from multiple histories and libraries, and from within dataset collections. This greatly reduces the need for users to copy datasets between different components of Galaxy, and makes it easier and faster for users to find datasets for analysis.

### Interactive tools for exploratory data analysis

Analysis of any biomedical data occurs in several distinct modes. For primary processing at the start of an analysis, there are well established practices and corresponding tools for data quality assessment and initial steps. In sequence-based experiments, these may be read mapping, variant identification, peak prediction and so on. Each of these steps has a well defined set of software tools that fit well into the Galaxy paradigm. However, research-focused data analysis invariably comes to a point where specialized tools no longer exist, and further result interpretation needs to be done on an *ad hoc* exploratory basis. This stage does not fit well into the historical Galaxy way of conducting analysis. In fact, tools designed specifically for exploratory data analysis, such as Jupyter (3) and RStudio (4), are often better choices for these modes.

**Figure 3.**
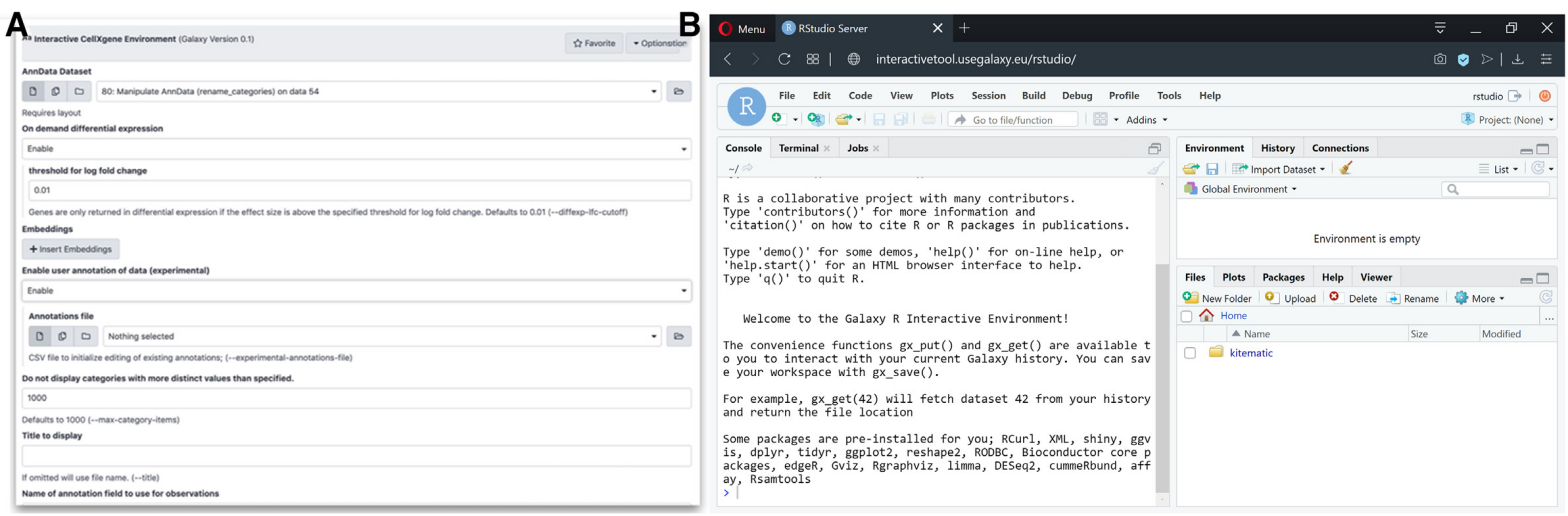
Example of an Interactive Galaxy Tool (IGT) for exploratory data analysis. The tool uses the same configuration file format as any other Galaxy tool (see Supplementary Figure S1). After processing single-cell RNA-seq data in Galaxy, users run the cell × gene tool through a standard Galaxy tool interface (panel **A**). Users can then use the tool to interactively explore their data, and perform server-side analysis on-the-fly. For instance, panel **B** shows RStudio launched within the IGT framework from the usegalaxy.eu server.

To address this challenge we have developed *Interactive tools*, which enables visualization and analysis environments such as Jupyter, RStudio and others to be used in Galaxy (see http://live.usegalaxy.eu). Starting an interactive tool is no different from invoking any other Galaxy tool, and inherits all the capabilities of existing Galaxy tools (Figure 3). *Interactive tools* succeed *interactive environments* (5) as Galaxy's method for running open-ended visualization and analysis environments, making them more robust and usable.

### Federated identity and access management

A growing number of well-curated and essential biomedical data repositories now exist. In accordance with access control regulations, some repositories authorize only users with verifiable identities to access their data. OpenID Connect (OIDC) protocol is a leading web standard for user identification and securely sharing identities between applications. We have recently enabled user authentication through the OIDC protocol and users can now securely login to Galaxy using their existing social and institutional identities (6).

Leveraging the OIDC protocol, we have also implemented authorization delegation, where users can securely grant Galaxy authorization to access their private resources in the Cloud. Using this approach, authorization is based on short-lived tokens generated specifically for a Galaxy instance and are independent from any user's authorization. The tokens cannot be exploited to impersonate the instance or the user, and their authorization scope follows the principle of least privileges and can be modified or revoked at any time (6).

### Access to user-owned cloud-based storage

Cloud computing platforms provide reliable and cost-effective data storage solutions. We have implemented features that enable users to transfer data between Galaxy and cloud-based storage services including Amazon Simple Storage Service (S3), Microsoft Azure, and Google Cloud Storage. Users can now directly copy data stored on the Cloud to a Galaxy sever as well as copy data from Galaxy onto the Cloud. Previously, to copy data between Galaxy and the Cloud, users were required to either first download data to local storage and then upload to Galaxy or the Cloud, or to use *signed URLs* in order to copy from Cloud to a Galaxy *history*. These approaches have some drawbacks; for instance, obtaining a *signed URL* for data stored on Cloud requires platform knowledge, and using local storage as cache to transfer data between Galaxy and cloud resources can be insecure and tedious for large data sizes. However, with the new feature, users grant Galaxy authorization to access their cloud-based storage through a secure approach (6), and data is transferred directly from a cloud storage service to a Galaxy server.

### Increasing accessibility and utilization of compute infrastructure

**Figure 4.**
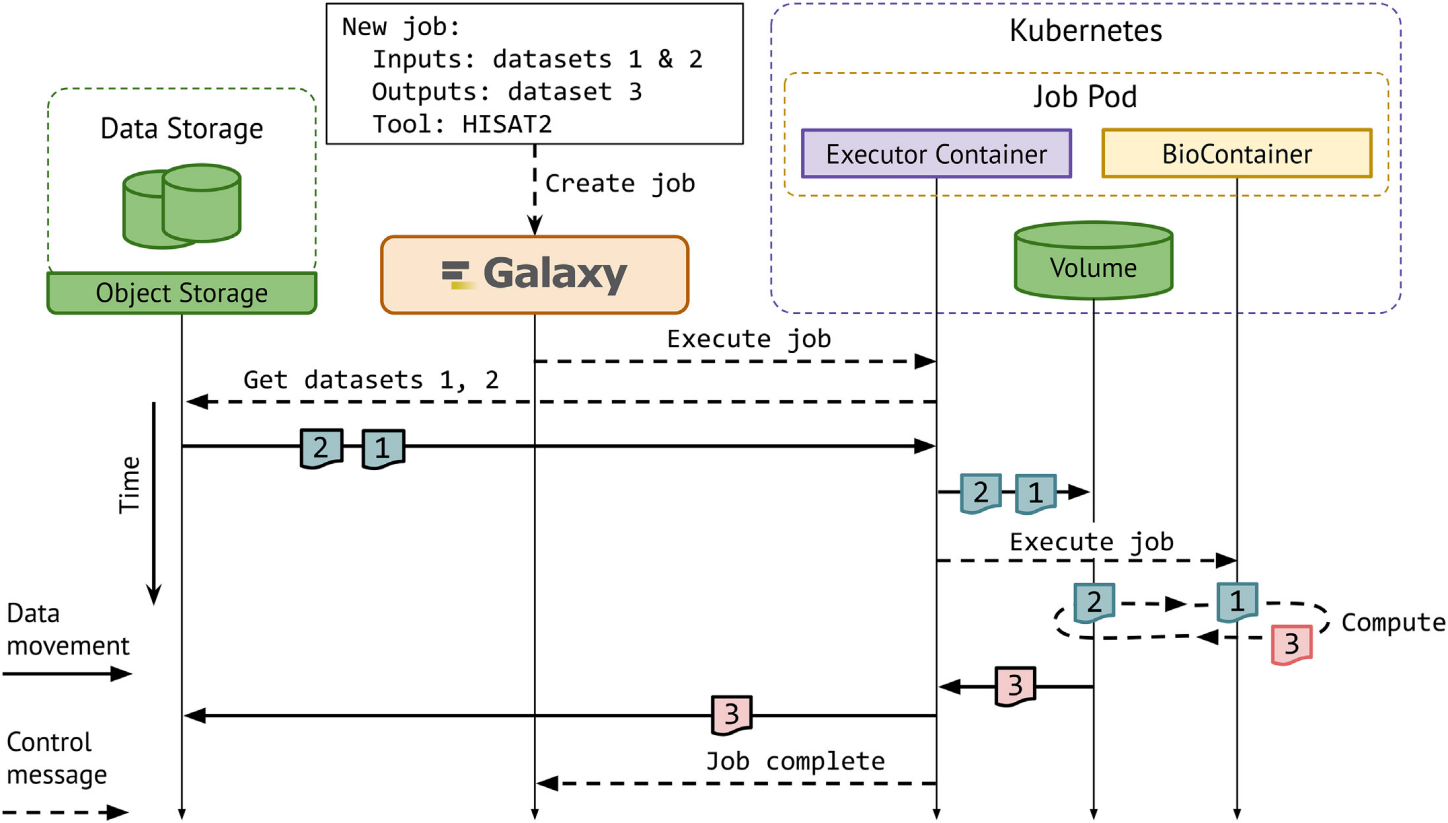
Streamlined data flow for isolated user jobs. As a user submits a job to Galaxy, Galaxy schedules the job as a self-contained, isolated unit passing only metadata about the job inputs (which can be a collection of datasets). Each job will communicate with the relevant data store to retrieve input data, execute the job, and persist the data. This method enables a more secure and efficient job execution.

The ever increasing computational demands of biomedical data analysis has been a driving motivation for a continuous improvement in how Galaxy utilizes heterogeneous compute infrustructures. Natively, Galaxy runs user analysis on compute resources where data is shared between Galaxy and the resources via a single shared file system. However, this requirement limits where Galaxy can be installed, and to alleviate it, we have developed Pulsar—an application that runs on remote computers (e.g. (7–9) as used by usegalaxy.org), listens to Galaxy *job* execution requests (a *job* is a unit of execution that runs a given tool on given input data), automatically transfers the *job* data, and executes the *job*. However, this process involves two data transfers: first Galaxy fetches input data from its persistent storage, then sends it to the remote computer where Pulsar executes the *job*. To optimize this process, we enhanced the implementation to enable Pulsar to directly fetch the data and hence reduce to one data transfer (see Figure 4). This also paves a path for Pulsar to fetch data directly from a user's private storage, such as an Amazon S3 bucket, without the Galaxy server's involvement; this feature is being actively developed.

### Workflow, scheduling, pipeline comparison, and parameter sweeps

Galaxy's workflow engine has been improved for efficient scheduling of complex workflows involving thousands of datasets and supporting a wide range of dataset relationships and collections. The consistency of the workflow editor has been improved to support non-file step parameter nodes and non-data outputs. The Galaxy workflow language has been significantly improved, with a new YAML-based format offering better readability and portability.

### New and updated tool suites

Several new tool suites have been integrated into Galaxy to reflect new analysis needs of the biomedical community, with each suite encompassing tools required to perform an end-to-end analysis of data generated by a particular technology. For analysis of single-cell datasets, the Seurat (10,11) and Scater (12) toolkits have been integrated into Galaxy. Tools in these suites enable complete analysis of single-cell data, from quality control to clustering and visualization. An ensemble of machine learning tools has also been added to Galaxy (https://usegalaxy-eu.github.io/index-ml); leveraging Scikit-learn (13) and TensorFlow (14), users can build predictive models from labeled datasets in Galaxy. These tools include approaches for data preprocessing (e.g. normalization), feature selection, defining and training regression and classification models using both traditional and deep learning approaches, model stacking to create meta-ensembles, and methods for evaluating model performance. Image analysis tools have also been added to Galaxy, with applications in chemoinformatics and histopathology. These tools make ImageJ (15,16) features available via Galaxy.

To facilitate particular analyses, we have deployed domain-specific Galaxy instances that provide users with domain-specific preconfigured workflows, tool suites, and interactive training materials. These instances simplify use of Galaxy for specific applications, such as metabolomics (https://metabolomics.usegalaxy.eu), metagenomics (https://metagenomics.usegalaxy.eu), proteomics (https://proteomics.usegalaxy.eu), and single-cell omics (https://singlecell.usegalaxy.eu).

## GALAXY GLOBAL ECOSYSTEM

Galaxy has grown into a thriving global ecosystem supported by a vibrant community of biomedical researchers, tool developers, and system engineers. This coherence is realized by a multitude of components including a public repository of all 7500+ tools integrated into Galaxy, named ToolShed (1), a public repository of over 36,000 Singularity containers (17) for all Bioconda packages (18) that provide the software dependencies for Galaxy tools, and 5.6TB (compressed) of publicly available reference and index data. Leveraging these resources, Galaxy instances running on institutional computing clusters, cloud platforms, and even the https://usegalaxy.org server itself, all see a unified and consistent view of all of the shared components. When a Galaxy instance anywhere in the world needs to run an analysis, it can fetch the required reference or index data and a container with the appropriate tool from these repositories and use them to execute the analysis—ensuring a common availability across all Galaxy instances. We have continued developing features to improve this coherence; namely, we are developing features to ensure that workflows can be shared between different Galaxy instances and still execute correctly and reproducibly. The three Galaxy instances with largest user bases, i.e. https://usegalaxy.org (hosted in U.S.), https://usegalaxy.eu (hosted in Europe), and https://usegalaxy.org.au (hosted in Australia), all use these shared resources.

## COMMUNITY

The Galaxy Community (https://galaxyproject.org/community) is vital to the success of the project, and this is reflected throughout the Galaxy ecosystem.

### Support and communication

In late 2018, we moved our online help forum to a new platform (https://help.galaxyproject.org/). Previously, our primary support channel had been a Biostars-based forum, and a mailing list before that. This move has been popular, with over 1200 accounts and 1100 threads created in the first 15 months, and an average of 488 page views per day. The Galaxy community now also provides support via Gitter (a developer-oriented chat room, available from https://gitter.im/galaxyproject/) with 1460 contributors.

Galaxy provides news and announcements through several channels. The *Galaxy Community Hub*, now in its fourth year, includes news items, blog posts, and an events calendar. Monthly newsletters are posted to the *hub* and highlight upcoming events, open positions, relevant news items, and recent releases and platform news. The newsletters and other significant items are announced on the Galaxy-Announce mailing list (with over 11 000 members) and Twitter (with over 9000 followers).

There are different ways to use Galaxy. To help users choose a method that suits their application best, we launched the *Galaxy Platform Directory* (https://galaxyproject.org/use/) in 2018. This is a searchable directory that lists ways to easily use Galaxy, either immediately (on over 125 public Galaxy servers), or after setup (with 5 commercial Cloud instances, 30 containers, 6 virtual machines or 12 academic cloud providers).

### Communities, events and training

The Galaxy Community supports many thriving regional communities which contribute code to the software framework based on their needs, run Galaxy instances for their users, and hold local meetings and training events. Europe and Australia are the largest examples, each with regional events and training. Other communities include a pan-African group, groups in France, Netherlands, and Japan. In 2020, we welcome new groups in India, Southeast Asia, and Korea.

The community organized 261 events with Galaxy-related content in 2018–2019 (https://galaxyproject.org/events/). That includes 164 training events, 83 conferences or meetings, and 17 collaborative work events, with Galaxy as the focus of 131 of those events. The events were held in 31 different countries, and were organized primarily by local Galaxy community members.

The *Galaxy Training Network* (GTN, https://training.galaxyproject.org/) provides a library of slides, hands-on-tutorials, and training datasets covering many domains in biomedical research. It also includes tutorials on how to administer Galaxy servers, wrap tools for Galaxy, and contribute code to the Galaxy software framework and to the GTN library itself. The GTN is a community managed and driven effort that has become the driving force behind the large number of training events that now use Galaxy. The GTN now contains 155 tutorials in 20 topic areas (e.g. transcriptomics and proteomics), created by 146 contributors. In addition, the European team has implemented Training Infrastructure as a Service (TIaaS), which provides instructors with reserved resources on https://usegalaxy.eu for the duration of training, and supports instructor tracking of trainee's progress.

The *Intergalactic Utilities Commission* (IUC) is another community driven group charged with establishing and maintaining best practices and gold-standard tool wrappers for the Galaxy ToolShed (1). The ToolShed now contains over 7500 tool definitions, created by over 600 unique contributors.

We continue to support regional meetings and to hold an annual *Galaxy Community Conference* (GCC) for the global community. GCC has run every year since 2010, and has had 200 or more participants since 2012. In 2018, and again in 2020, we colocated GCC with the *Bioinformatics Open Source Conference* (BOSC). These events feature training days, a multi-day conference and several days of collaborative work. GCC brings together people from all over the world to establish and reinforce connections in the community. In 2020, we are holding this event online, and in both the eastern and western hemispheres.

The last two years have seen an increase in our outreach to researchers in underrepresented groups. Galaxy has recently had workshops and speakers at the *Society for Advancement of Chicanos/Hispanics and Native Americans in Science* (SACNAS 2017–19), and *American Indian Science and Engineering Society* (AISES 2019); have offered travel fellowships for GCCBOSC 2018, GCC2019, Galaxy Africa 2018, Galaxy Admin Training 2020; and offered childcare at GCCBOSC 2018 and GCC2019.

### Publications using galaxy

The project tracks publications that use, reference, extend or implement Galaxy. In 2020 we reached over 9000 total publications, including over 7,500 journal articles, 500 books, 400 conference papers, and 300 theses (https://www.zotero.org/groups/galaxy). Over 5000 of these publications cited Galaxy in their methods. This publication corpus reflects the broad range of domains that Galaxy is applied to, including life science domains beyond genomics (e.g. ecology, proteomics), as well as domains outside the life sciences (e.g. natural language processing, climate science).

## Supplementary Material

gkaa434_Supplemental_File
