# The Galaxy platform for accessible, reproducible and collaborative biomedical analyses: 2020 update

**DOI:** 10.1093/nar/gkaa554

**Published:** 2020-06-25

**Authors:** Vahid Jalili, Enis Afgan, Qiang Gu, Dave Clements, Daniel Blankenberg, Jeremy Goecks, James Taylor, Anton Nekrutenko

**Affiliations:** Department of Biomedical Engineering, Oregon Health & Science University, Portland, OR, USA; Department of Biology, Johns Hopkins University, Baltimore, MD, USA; Department of Biomedical Engineering, Oregon Health & Science University, Portland, OR, USA; Department of Biology, Johns Hopkins University, Baltimore, MD, USA; Genomic Medicine Institute, Lerner Research Institute, Cleveland Clinic, Cleveland, OH, USA; Department of Biomedical Engineering, Oregon Health & Science University, Portland, OR, USA; Department of Biology, Johns Hopkins University, Baltimore, MD, USA; Department of Biochemistry and Molecular Biology, Penn State University, University Park, PA, USA

*Nucleic Acids Research*, 2020, gkaa434, https://doi.org/10.1093/nar/gkaa434

Galaxy project is a community driven effort to promote openness and reproducibility of data analyses in biomedical research. Our original publication has made a grave mistake of omitting many key participants of this effort. Here we are attempting to correct this error. We are including a new, greatly expanded version of Table 1. Again, we regret our mistake and will ensure that it will not occur again.

**Table 1.** Members of the four major regional Galaxy teams–Africa, Australia, Europe and North America

Region	Members	Affiliation
Africa	Christopher Barnett, Tharindu Senapathi	Chemistry Department and Scientific Computing Research Unit at the University of Cape Town
	Thoba Lose, Ziphozakhe Mashologu, Peter van Heusden	South African National Bioinformatics Institute, University of the Western Cape, South Africa
Australia	Catherine Bromhead, Simon Gladman, Nuwan Goonasekera, Christina Hall, Andrew Lonie	Melbourne Bioinformatics, University of Melbourne, Melbourne, Victoria, Australia
	Maria Doyle	Peter MacCallum Cancer Centre, Melbourne, Victoria, Australia
	Thom Cuddihy, Igor Makunin, Gareth Price, Nick Rhodes, Michael Thang	QFAB Bioinformatics, QCIF, Brisbane, Queensland, Australia
Europe	Loraine Brillet-Guéguen, Gildas Le Corguillé	ABiMS, Roscoff, France
	Christophe Antoniewski	ARTbio, CNRS and Sorbonne Université, Paris France
	Léa Bellenger	ARTbio, INSERM and Sorbonne Université, Paris, France
	Naïra Naouar	ARTbio, Sorbonne Université, Paris, France
	Nadia Goué	AuBi, Mesocentre, Clermont Auvergne University, France
	Saskia Hiltemann, Youri Hoogstrate, Bas Horsman, Rick Jansen, Yunlei Li, Andrew Stubbs, David van Zessen	Bioinformatics, Erasmus MC Cancer Institute, Rotterdam, Netherlands
	Frederik Coppens, Bert Droesbeke, Ignacio Eguinoa, Michiel Van Bel	Center for Plant Systems Biology, Vlaams Instituut voor Biotechnologie, Ghent, Belgium
	Jean-François Dufayard, Maryline Summo	CIRAD, Montpellier, France
	Anshu Bhardwaj	CSIR-Institute of Microbial Technology, France
	Tomas Klingström	Department of Animal Breeding and Genetics, Swedish University of Agricultural Sciences, Uppsala, Sweden
	Federico Zambelli	Department of Biosciences, University of Milan, Milano, Italy
	Rolf Backofen, Bérénice Batut, Simon Bray, Gianmauro Cuccuru, Anika Erxleben, Stephan Flemming, Björn Grüning, Alireza Khanteymoori, Anup Kumar, Jan Leendertse, Wolfgang Maier, Helena Rasche, Mehmet Tekman, Joachim Wolff, Oleg Zharkov	Department of Computer Science, Albert-Ludwigs-University Freiburg, Freiburg, Germany
	Anne Fouilloux	Department of Geosciences, University of Oslo, Norway
	Florence Combes, Yves Vandenbrouck	Department of Health, CEA, Grenoble, France
	Nicola Soranzo	Earlham Institute, Norwich Research Park, Norwich, UK
	Lucille Lopez-Delisle	EPFL SV ISREC UPDUB, 1015 Lausanne, Switzerland
	Pablo Moreno	European Bioinformatics Institute (EMBL-EBI)
	Hans-Rudolf Hotz	Friedrich Miescher Institute for Biomedical Research, Basel, Switzerland
	Sarah Maman	GenPhySE, Université de Toulouse, INRA, INPT, ENVT, Castanet Tolosan, France
	Matthias Bernt	Helmholtz Centre for Environmental Research, UFZ, Young Investigators Group Bioinformatics and Transcriptomics, Leipzig, Germany
	Anthony Bretaudeau	IGEPP, INRAE, Institut Agro, Univ Rennes, Rennes, France
	Timothy Dudgeon	Informatics Matters Ltd.
	Olivier Inizan, Valentin Loux	INRAE, Jouy-en-Josas, France
	Kenzo-Hugo Hillion, Valentin Marcon, Fabien Mareuil, Hervé Ménager, Rémi Planel	Institut Pasteur, Paris, France
	Marco Antonio Tangaro	Institute of Biomembranes, Bioenergetics and Molecular Biotechnologies, National Research Council, Bari, Italy
	Alexis Dereeper	Institute of Research for Development, Marseille, France
	Melanie Föll	Institute of Surgical Pathology, Medical Center, Albert-Ludwigs-University Freiburg, Freiburg, Germany
	Peter Cock	James Hutton Institute, UK
	Peter Selten	KWS SAAT SE & Co. KGaA
	Ruben Vorderman	Leiden University Medical Center, Netherlands
	Alan Amossé, Yvan Le Bras, Coline Royaux	Museum National d’Histoire Naturelle, Paris, France
	Franck Giacomoni	PFEM, INRAE, Saint Genès Champanelle, France
	Thanh Le-Viet, Andrew Page	Quadram Institute Bioscience, Norwich Research Park, Norwich, UK
	Thomas Lawson	School of Biosciences, University of Birmingham, UK
	Olivier Sallou	Univ Rennes, Inria, CNRS, IRISA, Rennes France
	Ralf Weber	University of Birmingham, UK
	Krzysztof Poterlowicz	University of Bradford, UK
	Ivan Kuzmin	University of Tartu, Estonia
North America	Dan Fornika	BC Centre for Disease Control, Canada
	Carrie Ganote	Bioinformatics Analyst at Indiana University, USA
	Dave Bouvier, Martin Čech, John Chilton, Nate Coraor, Assunta DeSanto, Jennifer Hillman-Jackson, Kaivan Kamali, Nick Keener, Delphine Lariviere, Anton Nekrutenko, Nick Stoler, Marius van den Beek	Department of Biochemistry and Molecular Biology, Penn State University, University Park, PA, USA
	Enis Afgan, Dannon Baker, Dave Clements, Sergey Golitsynskiy, Juleen Graham, Aysam Guerler, Mohammad Heydarian, Alexandru Mahmoud, Alex Ostrovsky, Nathan Roach, James Taylor, Jenn Vessio	Department of Biology, Johns Hopkins University, Baltimore, MD, USA
	Jeremy Goecks, Qiang Gu, Mason Houtz, Vahid Jalili, Luke Sargent	Department of Biomedical Engineering, School of Medicine, Oregon Health and Science University, Portland, OR, USA
	Michael Schatz	Dept. of Computer Science and Biology, Johns Hopkins University, Baltomore, MD, USA
	Daniel Blankenberg, Jayadev Joshi, Vijay Nagampalli	Genomic Medicine Institute, Lerner Research Institute, Cleveland Clinic, Cleveland, OH, USA
	Greg Von Kuster	Huck Institutes of the Life Sciences, Penn State University, University Park, PA, USA
	Robert Leach, Lance Parsons	Lewis-Sigler Institute of Integrative Genomics, Princeton University, USA
	Brad Langhorst	New England Biolabs, USA
	Philip Mabon, Aaron Petkau, Jeffrey Thiessen	Public Health Agency of Canada, Canada
	Arthur Eschenlauer, Tim Griffin, Pratik Jagtap, James Johnson, Praveen Kumar, Subina Mehta	University of Minnesota, Minnesota, Minneapolis, MN, USA

The published article has been updated. This correction does not affect the results or conclusions of the article.

