# Supplementary material for: The Galaxy platform for accessible, reproducible and collaborative biomedical analyses: 2020 update
Source: Nucleic Acids Res. 2020 Jun 1;48(W1):W395–402. doi: 10.1093/nar/gkaa434 (PMC7319590; doi:10.1093/nar/gkaa434)
Supplement: gkaa434_Supplemental_File [file gkaa434_supplemental_file.docx]

## Supplementary Material

Figure 1. An interactive Galaxy tool uses the same configuration file format as any other Galaxy tool.


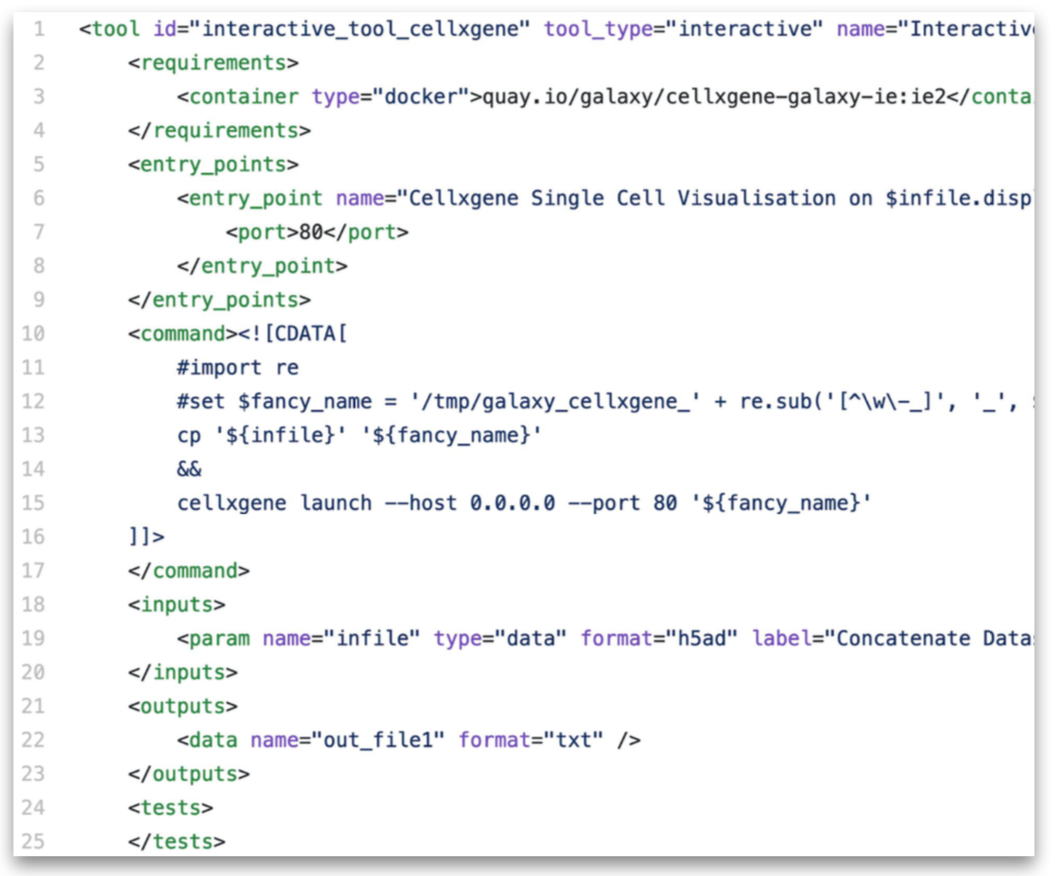


The rule used to create the collection displayed in Figure 2.

{

"rules": [

{

"type": "add_column_metadata",

"value": "hid"

},

{

"type": "add_column_metadata",

"value": "name"

},

{

"type": "add_column_regex",

"target_column": 1,

"expression": "(.*), .*",

"group_count": 1

},

{

"type": "add_column_regex",

"target_column": 1,

"expression": ".*, (.*)",

"group_count": 1

},

{

"type": "add_column_value",

"value": "#"

},

{

"type": "add_column_concatenate",

"target_column_0": 4,

"target_column_1": 2

},

{

"type": "add_column_concatenate",

"target_column_0": 4,

"target_column_1": 3

}

],

"mapping": [

{

"type": "group_tags",

"columns": [

2,

3

],

"editing": false

},

{

"type": "tags",

"columns": [

5,

6

],

"editing": false

},

{

"type": "list_identifiers",

"columns": [

1

],

"editing": false

}

]

}
